# Cortical excitation/inhibition ratios in patients with major depression treated with electroconvulsive therapy: an EEG analysis

**DOI:** 10.1007/s00406-023-01708-5

**Published:** 2023-11-10

**Authors:** Sven Stuiver, Julia C. M. Pottkämper, Joey P. A. J. Verdijk, Freek ten Doesschate, Eva Aalbregt, Michel J. A. M. van Putten, Jeannette Hofmeijer, Jeroen A. van Waarde

**Affiliations:** 1 Technical Medical Centre, Faculty of Science and Technology, Clinical Neurophysiology, University of Twente, Hallenweg 15, 7522NB Enschede, The Netherlands; 2 Department of Psychiatry, Rijnstate Hospital, Wagnerlaan 55, P.O. Box 9555, 6815AD Arnhem, The Netherlands; 3 Department of Neurology, Rijnstate Hospital, Wagnerlaan 55, 6815AD Arnhem, The Netherlands; 4 Department of Surgery, Amsterdam UMC Location Vumc, Boelelaan 1108, 1081HZ Amsterdam, The Netherlands

**Keywords:** Major depression, Electroconvulsive therapy, Excitation/inhibition ratio, Electroencephalography

## Abstract

Electroconvulsive therapy (ECT) is an effective treatment for major depression, but its working mechanisms are poorly understood. Modulation of excitation/inhibition (E/I) ratios may be a driving factor. Here, we estimate cortical E/I ratios in depressed patients and study whether these ratios change over the course of ECT in relation to clinical effectiveness. Five-minute resting-state electroencephalography (EEG) recordings of 28 depressed patients were recorded before and after their ECT course. Using a novel method based on critical dynamics, functional E/I (fE/I) ratios in the frequency range of 0.5–30 Hz were estimated in frequency bins of 1 Hz for the whole brain and for pre-defined brain regions. Change in Hamilton Depression Rating Scale (HDRS) score was used to estimate clinical effectiveness. To account for test–retest variability, repeated EEG recordings from an independent sample of 31 healthy controls (HC) were included. At baseline, no differences in whole brain and regional fE/I ratios were found between patients and HC. At group level, whole brain and regional fE/I ratios did not change over the ECT course. However, in responders, frontal fE/I ratios in the frequencies 12–28 Hz increased significantly (*p*_FDR_ < 0.05 [FDR = false discovery rate]) over the ECT course. In non-responders and HC, no changes occurred over time. In this sample, frontal fE/I ratios increased over the ECT course in relation to treatment response. Modulation of frontal fE/I ratios may be an important mechanism of action of ECT.

## Introduction

Major depression is one of the leading causes of disability world-wide, affecting over 160 million people in 2017 [1]. Although pharmacotherapy and psychotherapy are effective treatments, approximately 30% of the patients will subsequently develop treatment-resistant depression [2]. An effective and generally safe treatment for treatment-resistant depressed patients is electroconvulsive therapy (ECT) [3, 4]. Response rates of ECT vary from 64% in major depressed patients to 48% in case of antidepressants resistance [5]. The exact working mechanisms of ECT are poorly understood. Hypotheses point at diverse neurophysiological and neurobiochemical changes in the brain after ECT [6, 7]. Better understanding of the working mechanisms of ECT may ultimately contribute to better treatment outcome.

Nowadays, it is presumed that a proper balance between synaptic excitation (E) and inhibition (I) is essential for normal brain functioning and neuronal activity homeostasis [8, 9]. According to theoretical modeling, inputs of E and I are tightly balanced in pyramidal neurons, resulting in a relatively stable E/I ratio over both space and time [10]. Disturbances in the human brain’s E/I ratio probably play an important role in several neurodegenerative and neuropsychiatric disorders, including major depressive disorder (MDD) [11–14]. However, it is unclear whether all depressed patients show disturbed E/I ratios and whether such ratios represent ‘state’ or ‘trait’ conditions of the ‘depressed’ brain.

Measurements of E/I ratios in large populations of neurons in the human brain have been challenging. Recently, Bruining et al. developed a method to estimate E/I ratios non-invasively based on ongoing critical brain dynamics derived from electroencephalography (EEG) recordings [15]. By using the relationship between two characteristics of neuronal network activity (i.e., spectral power and long-range temporal correlations [LRTC]), a functional E/I (fE/I) ratio can be estimated. It was shown that this E/I measure was sensitive to pharmacological enhancement of gamma-aminobutyric acid (GABA)-A receptor-mediated inhibition, leading to decreased fE/I ratios in human EEG recordings [15]. Estimation of E/I ratios may provide new insights in the involved biological ‘state’ or ‘trait’ mechanisms in depressed patients. Also, estimating evolving E/I ratios over time may contribute to understanding neurophysiological (treatment) mechanisms. In this longitudinal cohort study, we estimate E/I ratios from resting-state EEGs in depressed patients before and after treatment with ECT. We study whether these ratios differ from those in healthy controls and whether the E/I ratios change during the ECT course in relation to treatment effectiveness.

## Methods

### Study populations

*Patients with major depression.* This is a post hoc analysis of data from depressed patients treated with ECT in Rijnstate Hospital (Arnhem, The Netherlands) and who participated in the StudY of effect of Nimodipine and Acetaminophen on Postictal Symptoms after ECT (SYNAPSE; NCT04028596). SYNAPSE is a randomized controlled trial with a three-condition cross-over design. Patients received nimodipine, acetaminophen, or a placebo (water) in random and counterbalanced order at a maximum of 2 h before each ECT session. The order of the treatment conditions was randomized and differed across patients [16]. Repeated EEG measures before, during, and until one hour after the ECT sessions were collected. Inclusion criteria for patients were age ≥ 18 years and having a current clinical diagnosis of major depression (i.e., classified as unipolar, bipolar, schizoaffective disorder in DSM-5). The local medical ethical committee approved the study protocol (NCT04028596) and all included patients provided oral and written informed consent.

*Healthy controls.* To correct for test–retest variability of fE/I ratios, we included a public EEG dataset of healthy controls (HC) with repeated EEG recordings at two different time points [17]. None of these participants reported psychiatric or neurological symptoms. HC were aged ≥ 18 years and provided oral and written informed consent [18].

### Electroconvulsive therapy

ECT was administered according to the standard treatment guidelines in The Netherlands [19], mostly twice weekly. The Thymatron System IV device (Somatics Incorporation Lake Bluff, Illinois, USA) was used to administer ECT stimuli with a constant-current (0.9 Ampère), bidirectional, square wave and brief pulse (1 ms). Electrodes were placed either unilateral (right [RUL]) or bi(fronto)temporal (BL). Dose titration or age-based dosage were used to choose the stimulus charge, which was adjusted during the course according to the psychiatrist’s discretion. In case patients did not improve after six sessions, unilateral placement was switched to BL during the ECT course. Anesthesia was mostly provided with etomidate (0.2–0.3 mg/kg body weight) for sedation and succinylcholine (0.5–1 mg/kg body weight) for muscle relaxation. Cessation of the ECT course was decided based on the treatment response, estimated by clinical judgement of the treating psychiatrist and clinically rated by using the Hamilton Depression Rating Scale (HDRS) [20].

### EEG data

*Patients with major depression.* Silver/silver chloride cup electrodes were placed on the scalp according to the International 10–20 system. To ensure enough space for placement of the ECT electrodes in patients receiving BL stimulation, EEG electrodes T3 and T4 were placed 10% behind and F7 and F8 above the pre-defined location. For patients receiving RUL stimulation, T4 and Cz were moved behind and F8 above. EEGs were recorded using a full-band DC amplifier (TMSi) and a NeuroCenter EEG recording system (Clinical Science Systems, Leiden). EEG recordings were sampled at 256 Hz. The impedance was kept below 5 kΩ. For the analyses, five-minute EEG recordings were used, measured with eyes closed prior to the first (or if missing, the second) ECT session (i.e., pre-ECT) and 2 weeks after the total ECT course (i.e., post-ECT). In case the 2-week post-ECT course measurement was missing (i.e., lost to follow-up), a resting-state EEG recording that was made before the last ECT session was used.

*Healthy controls*. Four-minute EEG recordings with eyes closed were used. EEG data were recorded using a 64-channel (silver/silver chloride) BioSemi ActiveTwo system (BioSemi B.V., Amsterdam) and were sampled at 1024 Hz [18], at two time points (i.e., T_1_ and T_2_). The available channels were reduced to the similar EEG electrodes of the patients. Here, EEG electrode T4 corresponded to T8, T3 to T7, T6 to P8, and T5 to P7.

### EEG pre-processing

Both EEG datasets were band-pass filtered (0.5–30 Hz; first-order Butterworth filter) and visually inspected for artifacts. Cz was used as reference electrode. EEG data of HC were resampled to 256 Hz. EEG electrodes containing (excessive) noise were rejected for analysis and segments containing artifacts were removed. This was considered justified, as removing segments from a signal and constructing the remaining together does not affect the scaling behavior of positively correlated signals [21]. All pre-processing steps and analyses were conducted with MATLAB R2022b (MathWorks, Natick, MA, USA).

### Spectral power

The power spectral density (PSD) of each EEG electrode pair was estimated using Welch’s method in five second artifact-free segments with an overlap of 50%. To compare PSD values, these were averaged in frequency bins of 1 Hz in the frequency range 0.5–30 Hz.

### Functional excitation/inhibition ratio (fE/I)

A requirement for estimating fE/I ratios was the existence of a co-variation between the amplitude of the spectral power and the fluctuation function. To ensure this, only data consisting of LRTC were used to estimate fE/I values. Detrended fluctuation analysis (DFA) was done to check for the existence of LRTC with DFA exponent > 0.60 as threshold [22]. When performing DFA, the fluctuation function is computed, which is plotted on logarithmic axes. The fluctuation function is expressed as

1 $$\langle F(t)\rangle =\mathrm{mean}(\sigma \left(W\right))$$

with $F(t)$ the fluctuation function and $\sigma$ the standard deviation of the detrended signal of a set windows ($W$) of separate time series of length $t$, with an overlap of 50%. The DFA exponent is the slope of the fluctuation function calculated using linear regression. After removing segments containing artifacts, the DFA exponent was fit between 2 and 25 s. DFA analysis was performed for each individual frequency band (i.e., bins of 1 Hz in the frequency range 0.5–30 Hz).

If the DFA exponent exceeded the threshold, a normalized fluctuation function nF(t) was computed by dividing each windowed signal profile by the mean amplitude of that window. Here, windows with a length of five seconds and an overlap of 80% were used. The fE/I ratio was defined as [15]

2 $$fE/I=1-{r}_{W\mathrm{amp},W\mathrm{nF}(t)}$$

with ${r}_{W\mathrm{amp},W\mathrm{nF}(t)}$ being the Pearson correlation between the set of windowed amplitude values (${W}_{\mathrm{amp}}$) and the set of detrended amplitude-normalized signal profiles (${W}_{\mathrm{nF}(t)}$). Hence, fE/I ratios below 1 indicated inhibition dominated networks, above 1 indicated excitation dominated networks, while balanced, critical networks would have a value of 1. fE/I ratios were estimated in frequency bins of 1 Hz in the frequency range 0.5–30 Hz.

### Statistical analyses

*Participant, treatment, and EEG characteristics.* After testing whether the data approximated a normal distribution (i.e., visualization of the histogram and Anderson–Darling test), we used mean ± standard deviation (SD) or median ± interquartile range (IQR) values to report participant, treatment, and EEG measures. In depressed patients, HDRS scores at baseline and after the ECT course were compared using the Wilcoxon signed-rank test. For spectral power analysis and estimation of fE/I ratios, we averaged all values over the available EEG electrodes (i.e., whole brain). Furthermore, to study regional effects on fE/I ratios three different regions were defined, i.e., the frontal region (including Fp1, Fp2, F3, F4, F7, F8, and Fz), centrotemporal region (including C3, C4, T3, and T4) and parieto-occipital region (including T5, T6, P3, P4, Pz, O1, and O2). For HC, we used the same descriptive statistics and EEG measures.

*Comparisons at baseline.* To test whether differences existed at baseline between fE/I ratios of patients (pre-ECT) and HC (T_1_), Wilcoxon rank-sum tests were performed.

*Changes over time.* Paired t-tests or Wilcoxon signed-rank tests were performed to test whether changes in spectral power and fE/I ratios occurred over the ECT course in patients (post-ECT–pre-ECT), as well as between the two time-point measurements in HC (T_2_–T_1_).

*Comparisons of responders and non-responders.* To study the association between changes in fE/I ratios and clinical outcome, patients were divided into the groups ‘responders’ (i.e., post-ECT HDRS score decrease ≥ 50% compared to baseline) and ‘non-responders’ after ECT. Next, these groups were analyzed regarding baseline EEG measures (pre-ECT) and changes in fE/I ratios over the ECT course (post-ECT–pre-ECT).

For all analyses, *p*-values < 0.05 were considered statistically significant. In case of multiple testing, also the false discovery rates (FDRs) were computed whereby *p*_FDR_ < 0.05 values were considered statistically significant.

## Results

### Participant, treatment, and outcome characteristics

We included a total of 28 patients with major depression (23 unipolar depressive disorder and five bipolar depressive disorder) and 31 HC. The SYNAPSE study consisted of 33 patients. Two patients were excluded from our current analysis, because they received maintenance ECT and therefore the HDRS scores were not suitable to study effectiveness of an index ECT course. Two patients were excluded, since they already received > 2 ECT sessions upon inclusion in SYNAPSE; therefore, their baseline EEGs were missing. One patient dropped out before the ‘end-measurement,’ two weeks after the ECT course, and therefore, the HDRS score was missing. In total, data from 28 ECT patients from the SYNAPSE trial were included in the current analyses. The original dataset of HC consisted of 41 participants with two measurements (mean 301 days ± 125 days SD in between), but 10 subjects had to be excluded because of poor data quality.

*Comparisons between patients and healthy controls.* Sex frequencies were equal in the patient group (*n* = 16 females; 57%) and the HC group (*n* = 18 females; 58%; χ^2^ = 0.0051; df = 1; *p* = 0.94). The patient group appeared significantly older (median = 54 years; IQR = 23) compared to HC (median = 39 years; IQR = 15.8; *p* < 0.001). Patients received a median of 12 ECT sessions (IQR = 6) during the ECT course and 20 (71%) were treated with BL stimulation. HDRS scores decreased significantly after the ECT course (median HDRS score pre-ECT = 25; IQR = 10; median HDRS score post-ECT = 12; IQR = 11; *p* < 0.001). Half of the patients reached the criterion of responder after the ECT course (*n* = 14; 50%) and seven (25%) reached complete remission (i.e., HDRS score post-ECT ≤ 7). An overview of the characteristics and comparisons is shown in Table 1.

**Table 1:** Clinical and demographic characteristics of patients with major depression and healthy controls

	Patients with major depression (*n* = 28)	Healthy controls (*n* = 31)	Between-Group comparisons
Age (IQR)	54 (23)	39 (15.8)	< 0.001^a^
Sex (M/F)	12/16	13/18	0.94^b^
Pre-HDRS score (IQR)	25 (10)	–	–
Post-HDRS score (IQR)	12 (11)^c^	–	–
Electrode placement (BL/RUL)	20/8	–	–
Total number of ECT sessions (IQR)	12 (6)	–	–

IQR = interquartile range; M = male; F = female; HDRS = Hamilton Depression Rating Scale; ECT = electroconvulsive therapy; BL = bi(fronto)temporal; RUL = right unilateral; ^a^Wilcoxon rank-sum test, *p*-value; ^b^χ^2^, *p*-value; ^c^ = Wilcoxon signed-rank test, *p* < 0.001

*Comparisons between responders and non-responders*. No differences in age were found between responders (median = 61 years; IQR = 20) and non-responders (median = 46 years; IQR = 21; *p* = 0.08). Sex frequencies were equal in both groups (χ^2^ = 0; df = 1; *p* = 1). The total number of ECT sessions did not differ between responders (median = 12; IQR = 6) and non-responders (median = 12; IQR = 5; *p* = 0.69). No differences in concomitant medication use (i.e., antidepressants [χ^2^ = 0.16; df = 1; *p* = 0.69], benzodiazepines [χ^2^ = 0.58; df = 1; *p* = 0.45], and antipsychotics [χ^2^ = 0.16; df = 1; *p* = 0.69]), and electrode placement ([χ^2^ = 2.80; df = 1; *p* = 0.09) between responders and non-responders were found. An overview of the characteristics and comparisons is shown in Table 2.

**Table 2:** Clinical and demographic characteristics of responders and non-responders after electroconvulsive therapy (ECT)

	Responders (*n* = 14)	Non-responders (*n* = 14)	Between-Group comparisons
Age (IQR)	61 (20)	46 (21)	0.08^a^
Sex (M/F)	6/8	6/8	1^b^
Total number of ECT sessions (IQR)	12 (6)	12 (5)	0.69^a^
Electrode placement (BL/RUL)	8/6	12/2	0.09^b^
Antidepressants (yes/no)	9/5	10/14	0.69^b^
Benzodiazepines (yes/no)	9/5	7/7	0.45^b^
Antipsychotics (yes/no)	10/4	9/5	0.69^b^

Responders = Decrease in Hamilton Depression Rating Scale score ≥ 50% compared to baseline; IQR = interquartile range; M = male; F = female; ECT = electroconvulsive therapy; BL = bi(fronto)temporal; RUL = right unilateral; ^a^Wilcoxon rank-sum test, *p*-value; ^b^χ^2^, *p*-value

### Spectral power

**Fig. 1:**
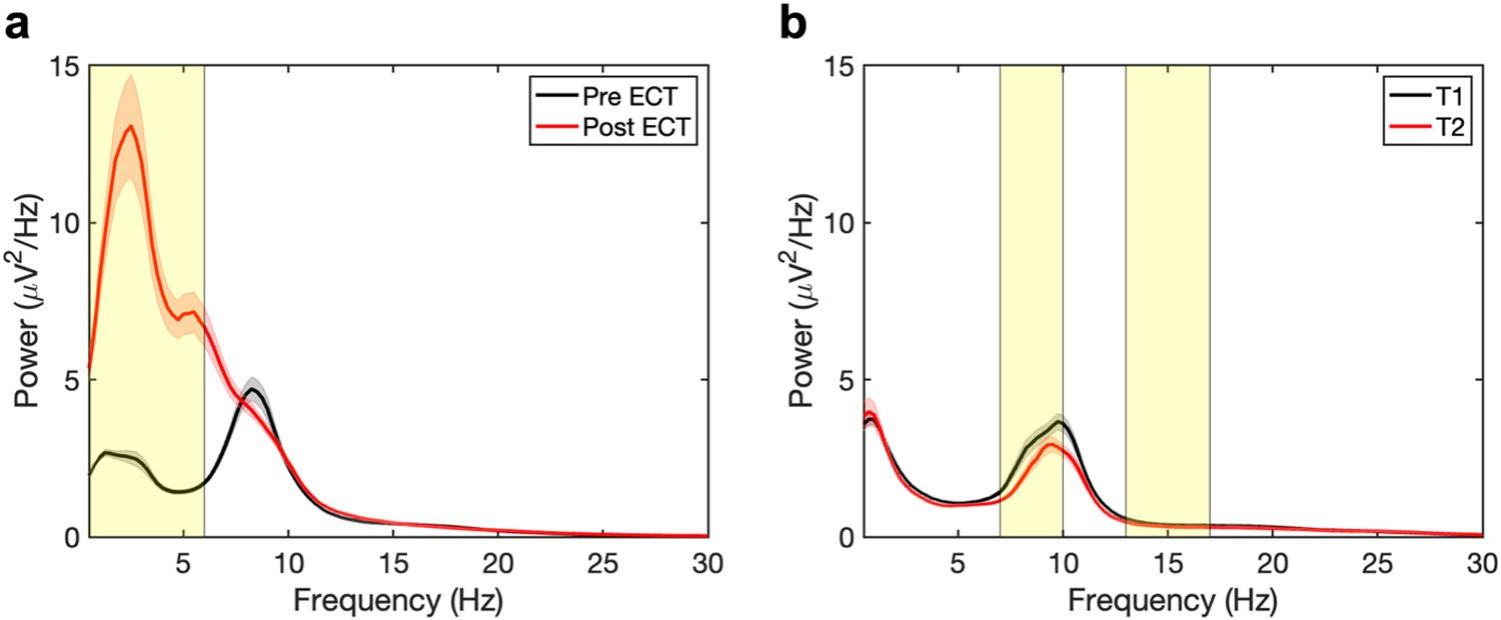
Averaged EEG power spectral density (PSD) values with standard error in patients with major depression **a** and healthy controls (HC) with eyes closed **b**. **a** In patients, PSD values increased in the frequency range 0.5–6 Hz (yellow box) over the electroconvulsive therapy (ECT)-course (*p*_FDR_ < .05); pre-ECT PSD (black line) and post-ECT PSD (red line). **b** In HC, PSD values at T_1_ (black line) in frequencies 7–10 Hz and 13–17 Hz (yellow boxes) were increased (*p*_FDR_ < 0.05) compared to T_2_ (red line). FDR = false discovery rate

In patients, after the ECT course (Fig. 1a, red line), PSD values between 0.5 and 6 Hz were increased (*p*_FDR_ < 0.05) compared to baseline (black line). No changes were observed for other frequencies. In the HC group, there were differences (*p*_FDR_ < 0.05) in PSD values in frequencies 7–10 Hz and 13–17 Hz between time points T_1_ (Fig. 1b, black line) and T_2_ (red line).

### Whole brain fE/I ratios

**Fig. 2:**
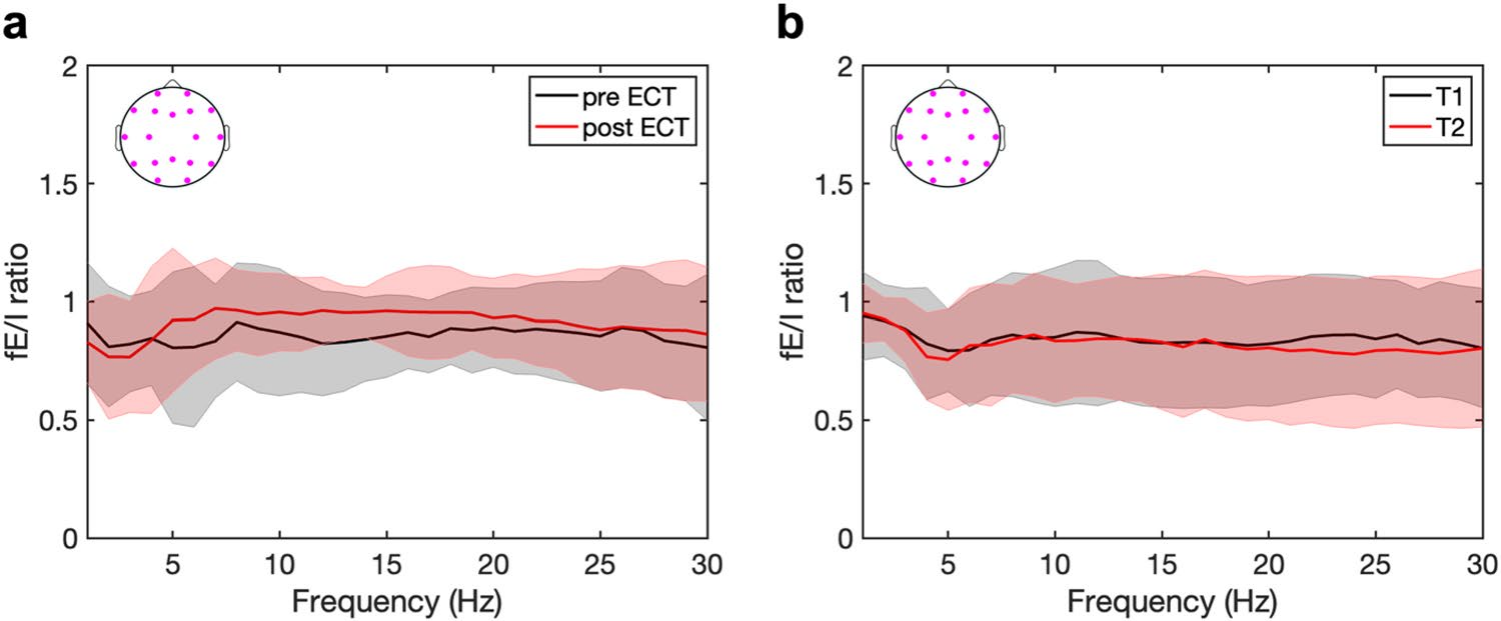
Whole brain functional excitation/inhibition (fE/I) ratios do not change in patients with major depression receiving electroconvulsive therapy (ECT) and in healthy controls (HC) with repeated measures. **a** In patients, no changes in fE/I ratios occurred over the ECT course, pre-ECT (black), and post-ECT (black). **b** In HC, fE/I ratios did not differ when measured at two different time points, T_1_ (black) and T_2_ (red). Solid lines and intervals represent median and interquartile range (IQR) values, respectively. Topoplots with purple dots indicate the electrode channels that were averaged

At baseline, no differences were found between whole brain fE/I ratios of patients (pre-ECT) and HC (T_1_). In Fig. 2a, the averaged fE/I ratios are shown before (solid black line) and two weeks after (solid red line) the ECT course. Whole brain analysis showed fE/I ratios close to value 1, estimated in the frequencies 0.5–30 Hz. No changes occurred in fE/I ratios over the ECT course in patients at group level. In HC, there were no differences between whole brain fE/I ratios estimated at the two different time points (i.e., T_1_ and T_2_), as is shown in Fig. 2b.

### Spatial fE/I ratios in patients and HC

At baseline, no differences were found between patients and HC regarding the averaged fE/I ratios in the frontal, centrotemporal, and parieto-occipital regions. In the total patient group after the ECT course, no changes in the three regional fE/I ratios occurred. Also, no differences in fE/I ratios in these three regions were found in HC between measurement T_1_ and T_2_.

### Spatial fE/I ratios in responders and non-responders

**Fig. 3:**
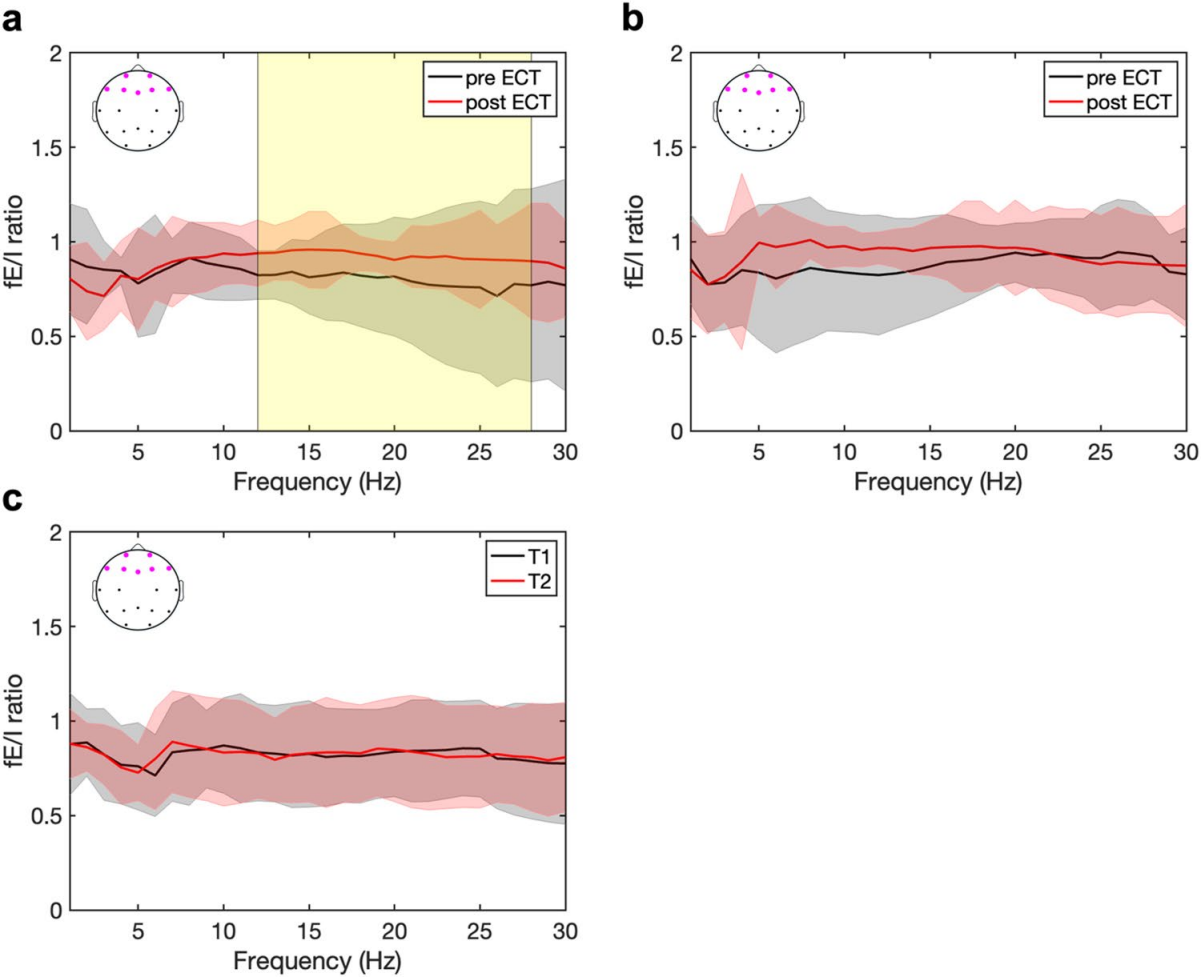
Frontal functional excitation/inhibition (fE/I) ratios increase in patients with major depression who respond to electroconvulsive therapy (ECT). **a** In responders, frontal fE/I ratios increased (yellow box [*p*_FDR_ < 0.05]) over the ECT course in the frequencies 12–28 Hz (i.e., beta rhythm). **b** In non-responders, no significant changes were found (*p*_FDR_ > 0.05); however, an increasing trend in frontal fE/I ratios over the ECT course in the frequencies 5–20 Hz was observed. **c** In healthy controls (HC), there were no changes between frontal fE/I ratios measured at time points T_1_ (black) and T_2_ (red). Solid lines and intervals represent median and interquartile range (IQR) values, respectively. Topoplots with purple dots indicate the frontal electrode channels that were averaged

At baseline (pre-ECT), no differences in whole brain and spatial averaged fE/I ratios were found between the groups of responders and non-responders (*p*_FDR_ > 0.05). In responders, frontal fE/I ratios increased over the ECT course in the frequencies 12–28 Hz (*p*_FDR_ < 0.05), as is shown in Fig. 3a. In non-responders, an increasing trend in fE/I ratios in the frequencies 5–20 Hz was observed, but no significant changes were found (Fig. 3b). Also, no changes occurred in HC between measurements T_1_ and T_2_ (Fig. 3c).

## Discussion

In this analysis of EEG data of 28 major depressed patients, treatment with ECT seemed to increase frontal fE/I ratios in the higher frequencies (i.e., 12–28 Hz). This effect was only observed in patients who responded to ECT, not in non-responders, while both groups did not differ in possible other determinants of ECT effectiveness, i.e., age, sex, concomitant medication use, or electrode placement. To our knowledge, this is the first study showing that ECT may modulate regional E/I ratios in relation to treatment response.

ECT induces a seizure in a patient’s brain (i.e., over-excitation), which is mostly followed by temporary strong postictal suppression of brain activity (i.e., over-inhibition). According to our observations, in patients responding to ECT, this treatment may assist neural networks in re-establishing a new balance between E and I. Lack of changes in fE/I ratios from repeated EEG measurements of HC strengthen our findings. The selective change of frontal fE/I ratios in responders may imply that effective ECT is associated with increase (toward more a balanced ratio) of frontal E/I ratios in depressed patients, which is in line with previous findings [23]. In other words, frontal fE/I ratios may represent a ‘state’ characteristic of the patients’ brains, which may change (or improve) through treatment.

Our findings add to the suggestions in the literature that ECT affects frontal regions of the brain which may be conditional for effectiveness [24]. Different structural and functional characteristics in frontal brain regions have been described in patients with MDD compared to HC. Based on neuro-imaging data, regional reductions in cortical gray matter in the frontal regions (i.e., medial orbitofrontal cortex and superior frontal gyrus) were found in patients compared to HC [25]. Also, in patients with MDD, increased functional connectivity was found between the subgenual anterior cingulate cortex (ACC) and medial temporal lobe compared to HC [26]. Therefore, alterations in the frontal brain region seem to be associated with major depression. However, in our EEG-study, at baseline, no differences in fE/I ratios were established between the depressed patients and HC, due to variations in fE/I ratios between patients. This may imply that fE/I ratios derived from the EEG are not suitable as biomarker for severity of depression or to predict effectiveness of ECT, yet. Nevertheless, changes in fE/I ratios within patients may indicate the effectiveness of an ECT course and may highlight an important working mechanism of ECT.

ECT may induce both structural and functional changes in the brain. Increases of gray matter volumes in the hippocampus, amygdala, and parts of the ACC after the ECT course have been reported, with mixed results regarding associations between these volume increases with effectiveness of ECT [27–29]. Functionally, increased ACC activity and increased hippocampal functional activity have been associated with ECT outcome [30, 31]. Our EEG findings may be in line with these previous magnetic resonance imaging (MRI) findings of (frontal) functional changes after ECT related to treatment outcome. Potentially, this and other EEG measures may capture underlying working mechanisms of ECT.

The increased spectral power that we found in the lower EEG-frequencies (i.e., f[0.5–6 Hz]) is consistent with previous findings of increased delta and theta activity after ECT [32, 33]. We speculate that these spectral changes are based on prolonged postictal slowing after the previous ECT sessions. We did not find changes in fE/I ratios in these lower frequency bands after ECT, showing that our observed changes in E/I ratios were not driven by the well-known spectral changes after ECT. In HC, variations in PSD values existed in the frequencies 7–10 Hz and 13–17 Hz, indicating that these values may vary within healthy subjects.

Currently, there is no consensus on the optimal way to estimate E/I ratios from human EEG recordings. We used resting-state EEG recordings to estimate fE/I ratios and—therefore—these reflect summations of many inputs, rather than subsets of only E or I inputs. Also, the fE/I ratios were estimated from spontaneous brain activity instead of evoked events. Although it has been suggested that these may largely agree [34], this is still uncertain. Different methods to measure E/I ratios from EEG recordings have been developed, such as the scaling exponent derived from the slope of the power law exponent of the PSD [35]. Although simple, robust, and capable of differentiation between states of awake and anesthesia, this method does not provide an absolute value indicating an increased, decreased, or balanced E/I ratio. Also, different frequency ranges to estimate the E/I ratio have been used and—at this moment—there is no consensus. Future studies are needed to compare different methods of estimating the E/I balance within the human brain, resulting in a consensus of what measure for E/I ratios is best to use.

The potential application of changing fE/I ratios during ECT as biomarker for effectiveness may not be limited to only ECT. Repetitive transcranial magnetic stimulation (rTMS) is an effective treatment for (major) depression [36] and may also affect E/I ratios. Besides in non-invasive stimulation techniques, E/I ratios may also change during pharmacological treatments that act on neuroreceptors (i.e., antidepressants, lithium carbonate, [es]ketamine). Therefore, estimating fE/I ratios during various treatments for depression may enhance our understanding of working mechanisms and contribute to individualization of treatment.

The critical brain dynamics theory states that neuronal networks operate near the critical point of a phase transition between a sub-critical (i.e., ordered) and supercritical (i.e., disordered) phase [38, 40, 41]. The brain is naturally poised near criticality and the primary parameter that is responsible for maintenance near this point (i.e., the controlling parameter) is the E/I ratio. It may therefore seem surprising that our estimated fE/I ratios had values slightly below 1 in our HC group (as well as in the depressed patients at baseline). According to the definition of fE/I, this indicates that the cortical networks act in (slightly) sub-critical regimes. In fact, this observation is in line with neural activity of HC in vivo, which was shown to operate slightly sub-critical (i.e., at the sub-critical border of the transition region) rather than at the critical point [42].

### Strengths and limitations

Strengths of this study include the use of paired EEG data from depressed patients before and after the ECT course and the use of a HC group to account for test–retest variability. The strength of the fE/I measure is that it enables real-time tracking of E/I ratios and it provides an absolute value indicating a balanced or unbalanced state [15]. However, some limitations are also present. First, patients were not restricted from taking medication (apart from acetaminophen, nimodipine, and non-steroidal anti-inflammatory drugs) during the ECT course, including antidepressants and benzodiazepines. Especially benzodiazepines are known to affect EEG activity, which may have influenced our results. During their ECT course, however, most patients did not switch in type nor dosage of the concomitant medications, which may have limited its effect on their evolution of fE/I ratios. Second, the effects of the used reference electrode (i.e., Cz) compared to reference schemes such as bipolar or common average on estimation of fE/I ratios is unknown. In other methods to estimate the E/I ratio, this has been shown to have profound effects, as it affects PSD values [43]. Our used method calculates a normalized fluctuation function, nF(t), that was computed by dividing each windowed signal profile by the mean amplitude of that window. Therefore, the effects of reference schemes on the fE/I ratios may be limited. Also, the E/I ratios are based on cortical activity, measured using EEG. Therefore, no (direct) information about E/I ratios of deeper structures is provided, which may also be affected in major depression or by ECT. The HC group was significantly younger compared to the ECT patients. This may have affected our results, as it is known that PSD values may decrease with age [44, 45]. Although the fE/I calculation method is based on a normalized fluctuation function, which may correct for age-related PSD changes, future studies should investigate the effect of age on fE/I ratios. Finally, the sample size of 28 depressed patients was rather small, which hampered proper analysis of confounders like age, sex, stimulation type and number of administered ECT sessions. Taking into account our less conservative control of the alpha error (i.e., FDR instead of Bonferroni correction), the other methodological limitations of this post hoc analyses, and our consideration of confounding variables (i.e., age, sex, total ECT sessions, electrode placement, and concomitant medications), we conclude with caution that the increase in frontal fE/I ratios in the frequency range 12–28 Hz may be a biomarker of positive ECT outcome. This finding needs replication in other samples and prospective studies.

## Conclusion

We found an increase in frontal E/I ratios (derived from resting-state EEG recordings) in severely depressed patients responding to ECT. In non-responders, no changes occurred and our results were corrected for test–retest variability by a healthy control group. Increase in frontal E/I ratios was not found at the whole brain level, pointing at a possible regional effect. The fE/I ratio may serve as reproducible parameter in future research. Recovery of frontal E/I ratios may indicate an important working mechanism of ECT.

## Data Availability

Individual participant data that underlie the results reported in this article, after de-identification (text, tables and figures) will be available including data dictionaries, as well as the study protocol and statistical analysis plan. Data will be available following article publication to researchers who provide a methodologically sound proposal to achieve aims in the approved proposal. Proposals should be directed to the principal investigator of SYNAPSE (jvanwaarde@rijnstate.nl). To gain access, data requestors will need to sign a data access agreement.
